# Improving women's diet quality preconceptionally and during gestation: effects on birth weight and prevalence of low birth weight—a randomized controlled efficacy trial in India (Mumbai Maternal Nutrition Project)^1^,^2^,^3^,^4^,^5^

**DOI:** 10.3945/ajcn.114.084921

**Published:** 2014-09-17

**Authors:** Ramesh D Potdar, Sirazul A Sahariah, Meera Gandhi, Sarah H Kehoe, Nick Brown, Harshad Sane, Monika Dayama, Swati Jha, Ashwin Lawande, Patsy J Coakley, Ella Marley-Zagar, Harsha Chopra, Devi Shivshankaran, Purvi Chheda-Gala, Priyadarshini Muley-Lotankar, G Subbulakshmi, Andrew K Wills, Vanessa A Cox, Vijaya Taskar, David JP Barker, Alan A Jackson, Barrie M Margetts, Caroline HD Fall

**Affiliations:** 1 From the Centre for the Study of Social Change, Mumbai, India (RDP, SAS, MG, HS, MD, SJ, AL, HC, DS, PC-G, PM-L, and GS); the Medical Research Council Lifecourse Epidemiology Unit (SHK, NB, PJC, EM-Z, AKW, VAC, DJPB, and CHDF), the National Institute for Health Research Southampton Biomedical Research Centre (AAJ), and Public Health Nutrition, Faculty of Medicine (BMM), University of Southampton, Southampton, United Kingdom; and Streehitakarini, Mumbai, India (VT).

## Abstract

**Background:** Low birth weight (LBW) is an important public health problem in undernourished populations.

**Objective:** We tested whether improving women's dietary micronutrient quality before conception and throughout pregnancy increases birth weight in a high-risk Indian population.

**Design:** The study was a nonblinded, individually randomized controlled trial. The intervention was a daily snack made from green leafy vegetables, fruit, and milk (treatment group) or low-micronutrient vegetables (potato and onion) (control group) from ≥90 d before pregnancy until delivery in addition to the usual diet. Treatment snacks contained 0.69 MJ of energy (controls: 0.37 MJ) and 10–23% of WHO Reference Nutrient Intakes of β-carotene, riboflavin, folate, vitamin B-12, calcium, and iron (controls: 0–7%). The primary outcome was birth weight.

**Results:** Of 6513 women randomly assigned, 2291 women became pregnant, 1962 women delivered live singleton newborns, and 1360 newborns were measured. In an intention-to-treat analysis, there was no overall increase in birth weight in the treatment group (+26 g; 95% CI: −15, 68 g; *P* = 0.22). There was an interaction (*P* < 0.001) between the allocation group and maternal prepregnant body mass index (BMI; in kg/m^2^) [birth-weight effect: −23, +34, and +96 g in lowest (<18.6), middle (18.6–21.8), and highest (>21.8) thirds of BMI, respectively]. In 1094 newborns whose mothers started supplementation ≥90 d before pregnancy (per-protocol analysis), birth weight was higher in the treatment group (+48 g; 95% CI: 1, 96 g; *P* = 0.046). Again, the effect increased with maternal BMI (−8, +79, and +113 g; *P*-interaction = 0.001). There were similar results for LBW (intention-to-treat OR: 0.83; 95% CI: 0.66, 1.05; *P* = 0.10; per-protocol OR = 0.76; 95% CI: 0.59, 0.98; *P* = 0.03) but no effect on gestational age in either analysis.

**Conclusions:** A daily snack providing additional green leafy vegetables, fruit, and milk before conception and throughout pregnancy had no overall effect on birth weight. Per-protocol and subgroup analyses indicated a possible increase in birth weight if the mother was supplemented ≥3 mo before conception and was not underweight. This trial was registered at www.controlled-trials.com/isrctn/ as ISRCTN62811278.

## INTRODUCTION

Low birth weight (LBW) is common in undernourished populations in low- and middle-income countries, predominantly because of intrauterine growth restriction, and is associated with increased perinatal mortality and childhood stunting, poorer childhood cognitive function, and increased adult chronic disease (1, 2). The WHO has set a global target to reduce LBW by 30% by 2025 (3).

Many trials have assessed the effect on birth weight of giving women micronutrient supplements during pregnancy. Systematic reviews have estimated that multiple-micronutrient supplementation by using either tablets or fortified drinks increases birth weight by 20–50 g and reduces LBW by 10–20% (4–6). An increase in dietary diversity has been advocated as a way of improving the micronutrient status in populations because it brings some benefits not provided by supplements (employment and economic gain for communities and possible additional nutrients) (7, 8). An observational study in Pune, India, showed that rural mothers who consumed foods rich in micronutrients (green leafy vegetables, fruit, and milk) more frequently had newborns who were heavier and larger in all body measurements (9). However, to our knowledge, no randomized studies have tested the effect of improving the mother's diet quality by using these foods.

For practical reasons, most previous supplementation trials have started after the onset of pregnancy, generally toward the end of the first trimester or later, thereby missing important processes in early pregnancy that influence fetal growth and later health and function such as periconceptional epigenetic changes, placentation, and organogenesis (10–12).

To test whether improving the mother's diet quality for a sustained period before and during pregnancy increases birth weight, we carried out a randomized controlled trial in low-income women in Mumbai, India. We created a snack that, when taken 3 d/wk in addition to the usual diet, increased women's average intakes of green leafy vegetables, fruit, and milk above the highest quartile in the Pune study (13) and compared it against a control snack made from vegetables of low micronutrient content. We recruited nonpregnant women who were intending to have children and planned to test the effect of starting supplementation ≥3 mo before pregnancy. The primary outcome was birth weight. The Pune study showed the strongest positive associations between diet and birth weight in thinner women, and we hypothesized a priori that there would be a larger intervention effect in women of lower BMI.

## SUBJECTS AND METHODS

### Setting and participants

The trial took place from January 2006 to May 2012 in the Bandra, Khar, Santa Cruz, and Andheri areas of the city of Mumbai, India, in slums covered by the health and social programs of the nongovernmental organization the Centre for the Study of Social Change (CSSC). Women were eligible if aged <40 y, married, nonpregnant, not sterilized, planning to have more children, and intending to deliver in Mumbai.

### Intervention

We considered various ways of supplying a daily, freshly prepared, safe, and palatable portion of food that contained green leafy vegetables, fruit, and milk to women who were living across an urban slum area ∼13 × 13 km. The best solution, after development and pilot testing in a different slum community (Shetanchowki, Mumbai), was snacks that resembled local street foods such as samosas and fritters, which could be filled with the key ingredients, cooked, packaged, and easily transported (13).

Treatment snacks contained fresh and dried green leafy vegetables, milk, and dried fruit (**Table 1**). Green leafy vegetables included spinach, colocasia, amaranth, fenugreek, coriander, shepu, spring onion stalk, and curry leaves. Initially, we used dried green leafy vegetables with the rationale being to provide more micronutrients per unit volume of green leafy vegetables. These vegetables were commercially produced, air-dried at room temperature, and supplied as powders or flakes. However, as the trial progressed, we increased the proportion of fresh leaves purchased from local markets, which improved the palatability without major changes in the nutrient content (**Table 2**). Dried fruits included figs, dates, raisins, mango, apple, gooseberry, and guava. Milk was included as commercially bought full-fat milk powder. Control snacks were made from low-micronutrient vegetables such as potato, tapioca, and onion, which were purchased from local markets. To avoid monotony for the women, we created 70 treatment and 40 control recipes from these foods of which 8–14 were in use at any time (*see* Supplemental Table 1 under “Supplemental data” in the online issue). Snacks were made fresh each day in a dedicated study kitchen at the CSSC. Both treatment and control snacks had similar added spices, bindings, and covering ingredients (wheat, rice, or chickpea flour and semolina) and (except for one recipe in each allocation group) were cooked by deep frying in sunflower oil.

**TABLE 1:** Ingredients of snacks at each stage of the trial*^1^*

	Treatment				
Ingredients	January 2006 to October 2006	October 2006 to June 2007	June 2007 to May 2012	January 2010 to May 2012 (fruit bar*^2^*)	Control: January 2006 to May 2012
Dry GLV powder (g)*^3^*	7.5	3.8	0	0	0
Milk powder (g)	16	12	12	0	0
Fruit powder (g)	4	4	0	0	0
Fresh GLV (g)*^3^*	0	29	30	0	0
Dried fruit (g)	0	0	4	60	0
Chickpeas (g)	0	0	0	2	0
Sesame seeds (g)	0	0	0	3	0
Low-micronutrient vegetables (g)*^4^*	0	0	0	0	18
Binding ingredients (g)*^5^*	30	28	30	0	22
Spices (g)	2	2	2	2	2

1 Treatment snacks were changed during the course of the trial to improve the palatability of snacks and, hence, 4 columns for treatment snacks are shown (*see* Intervention under Subjects and Methods). The nutrient content remained similar (Table 2). GLV, green leafy vegetable.

2 An uncooked fruit bar was introduced as a treatment snack once per week from January 2010. A sample recipe is shown in Supplemental Table 1 (under “Supplemental data” in the online issue).

3 GLVs included spinach, colocasia, amaranth, fenugreek, coriander, shepu, onion stalk, and curry leaves. Dried GLVs were air-dried at room temperature and supplied as powders or flakes.

4 Included potato and onion.

5 Binding ingredients used were wheat flour, rice flour, chickpea flour, or semolina.

**TABLE 2:** Nutrient composition and percentage contribution to nutrient requirements of snacks at each stage of the trial*^1^*

	Treatment				January 2006 to May 2012 (all snacks)	
January 2006 to October 2006	October 2006 to June 2007	June 2007 to May 2012	January 2010 to May 2012 (fruit bar*^2^*)	Treatment	Control
Micronutrient content/snack						
β-Carotene (RE)	114 ± 26*^3^*	200 ± 23	141 ± 85	353 ± 180	159 ± 55 (21–595)*^4^*	2 ± 1 (0–3)
Riboflavin (mg)	0.20 ± 0.01	0.21 ± 0.02	0.15 ± 0.03	0.04 ± 0.02	0.16 ± 0.04 (0.00–0.22)	0.01 ± 0.01 (0.00–0.02)
Folate (μg)*^5^*	26.0 ± 5.7	50.8 ± 19.5	67.5 ± 30.6	40.2 ± 35.9	58.5 ± 14.6 (5.2–93.0)	6.1 ± 4.6 (2.7–12.1)
Vitamin C (mg)	<1 ± 0.0	0.5 ± 0.6	2.1 ± 3.0	8.7 ± 12.7	2.1 ± 1.8 (0.0–36.6)	0.0 ± 0.0 (0.0–0.6)
Vitamin B-12 (μg)	0.64 ± 0.05	0.58 ± 0.16	0.31 ± 0.13	0.14 ± 0.15	0.38 ± 0.14 (0.00–0.74)	0.18 ± 0.25 (0.00–0.60)
Calcium (mg)	210 ± 14	275 ± 66	194 ± 35	76 ± 16	200 ± 42 (52–356)	25 ± 35 (8–87)
Iron (mg)	6.85 ± 1.07	5.90 ± 1.58	3.93 ± 1.26	1.75 ± 0.49	4.42 ± 1.27 (1.22–7.59)	0.90 ± 0.26 (0.65–1.28)
Macronutrient content/snack*^6^*						
Energy (MJ)	0.74 ± 0.09	0.70 ± 0.06	0.61 ± 0.07	0.92 ± 0.04	0.69 ± 0.08 (0.56–0.92)	0.37 ± 0.05 (0.27–0.66)
Protein (g)	7.3 ± 0.9	6.9 ± 0.7	6.4 ± 1.0	2.7 ± 0.3	6.4 ± 1.0 (2.7–7.9)	2.4 ± 0.6 (1.0–3.3)
Percentage of RNI*^7^*						
β-Carotene (RE)	14	25	18	44	20	<1
Riboflavin (mg)	14	15	11	3	11	<1
Folate (μg)	4	8	11	7	10	1
Vitamin C (mg)	<1	1	4	16	4	<1
Vitamin B-12 (μg)	25	22	12	5	15	7
Calcium (mg)	18	23	16	6	17	2
Iron (mg)	35	30	20	9	23	5

1 RE, retinol equivalents; RNI, reference nutrient intake.

2 An uncooked fruit bar was introduced as a treatment snack once per week from January 2010. A sample recipe is shown in Supplemental Table 1 (under “Supplemental data” in the online issue).

3 Mean ± SD (all such values).

4 Weighted mean ± SD; range in parentheses (all such values). The weighted average was based on the number of days that the snacks were distributed over the study period. The range is the lowest and highest nutrient contents measured in a sample of an individual snack.

5 Total folate.

6 Macronutrient content calculated from Indian Food Tables (14).

7 WHO/FAO recommended Reference Nutrient Intakes during the first trimester of pregnancy except for calcium for which only a third-trimester value was available (15).

We aimed to improve diet quality rather than specific nutrient intakes by raising intakes of green leafy vegetables, fruit, and milk. On average, treatment snacks contained 10–23% of the WHO/FAO recommended Reference Nutrient Intakes for β-carotene, riboflavin, folate, vitamin B-12, calcium, and iron (Table 2) (15). Snacks were tested approximately every 6 mo for micronutrient contents (Eclipse Ltd), and microbiological contamination (coliforms and aflatoxin; Intertek Testing Services) with consistently negative results.

### Recruitment and baseline investigations

The study area was divided into smaller areas that were based around 61 supplementation centers so that women would have to walk no further than 300–500 m from home to obtain their snacks. We used health clinics and offices of political parties, community organizations, and housing societies. Health workers known to families made home visits to explain the trial and deliver information leaflets. Community meetings were held to obtain community consent and answer questions. Recruitment camps were scheduled at which women were screened for eligibility, and individual written informed consent was obtained. Women's education and occupations were recorded. Socioeconomic status was assessed by using the Standard of Living Index on the basis of housing type, utilities, and household possessions (16). Tobacco use was recorded. Diet was assessed by using a food-frequency questionnaire (17). Weight and height were measured by using standardized techniques. Women were photographed for identity cards, which were color-coded to indicate the allocation group. The trial was initially anticipated to take 5 y; recruitment was prolonged until it was anticipated that we would have sufficient pregnancies and stopped in February 2011.

### Random assignment

Random assignment was purposively generated remotely in Southampton, United Kingdom. Women were individually randomly assigned, stratified by age, and BMI (3 groups for each). Because recruitment took place over several years, and we aimed to start supplementation quickly after recruitment to give as much time as possible on supplementation before pregnancy, random assignment was carried out in batches after every 1–3 recruitment camps. Initially, we used an SPSS (version 14.0; SPSS Inc) randomization function, which produced approximately equal groups. However, by April 2007, allocation groups differed in size by >100 subjects, and we changed to a similar procedure that incorporated a block-randomization program developed in house by using STATA software (version 12.1; StataCorp LP), which subsequently allocated exactly equal numbers to each group. Age and BMI stratification was identical for both methods.

### Blinding

Full blinding is not possible in a food-based trial. Treatment and control snacks were outwardly similar, but their contents looked different. To obscure allocation, we created 2 treatment and 2 control groups, each with an independent set of recipes. Four different snacks were produced daily in an unpredictable pattern. Staff who measured outcomes were blinded to the women's allocation groups. The 2 treatment groups and 2 control groups were merged for analysis.

### Supplementation

Snacks were produced daily except Sundays and public holidays, packaged in color-coded bags to match identity cards, and transported to supplementation centers by autorickshaw. Women were asked not to alter their usual diets, and snacks were available from 1500 to 1800 to interfere least with main meals. During Ramadan, when Muslims eat only between sunset and sunrise, the time was extended to 2000. Women were given 1 snack/d, and consumption was observed and recorded (1 = full; 0.5 = greater than or equal to half; 0 = less than half). Centre staff also recorded women's serial menstrual period dates. Compliance was defined as an average of ≥3 snacks/wk from 90 d before the last menstrual period date until delivery.

### Pregnancies

Women who missed 2 periods had a urinary pregnancy test, and if this test was positive, the women were invited to a central clinic at the CSSC at 9–13 wk of gestation for an obstetric assessment, hemoglobin measurement, and ultrasonography to confirm and date the pregnancy (18). Ultrasound scans were conducted by a single operator throughout the trial, and the sex of the fetus was never divulged to the parents; 51% of pregnancies were scanned before 12 wk, and an additional 21% of pregnancies were scanned before 20 wk. At 27–33 wk, we repeated the hemoglobin measurement and performed an oral-glucose-tolerance test (WHO protocol) (19). Samples were analyzed in a single laboratory. Apart from the previously mentioned investigations, which were carried out centrally by our research team, women continued to receive antenatal care from their own obstetricians and chose their place of delivery. Obstetricians generally prescribed iron (100 mg) and folic acid (500 μg) from the confirmation of pregnancy (20). If these supplements were not prescribed, or women were unable to afford them, we supplied the supplements free of charge. We did not assess compliance with these supplements. Women shown to have anemia or gestational diabetes were referred to their obstetricians for additional management. Women who opted for a termination of pregnancy made this decision in discussion with their obstetrician, and the research team played no role. Women continued to receive study snacks until delivery.

### Deliveries

Health workers were issued mobile phones, and families were asked to notify them when women went into labor. If women were not attending for supplementation, health workers visited the women 3 times/wk from 36 wk of gestation to maintain contact. Deliveries took place in 140 different institutions, ranging from small private nursing homes to large government hospitals. We aimed to measure newborns within 72 h of birth (achieved in 77% of births, treatment: 76% of births; control: 77% of births) but included measurements up to 10 d; the median (IQR) age at measurement was 45 h (24–81 h) [treatment: 47 h (25–84 h); control: 45 h (23–79 h)]. Trained research nurses measured weight (to the nearest 10 g; Seca scales; seca), crown-heel length to the nearest 0.1 cm, Rollameter; CMS Instruments), circumferences [occipitofrontal head, midupper arm, chest (xiphisternum level), abdomen (below the umbilicus)], and triceps and subscapular skinfold thicknesses. Circumferences were measured thrice to the nearest 0.1 cm by using fiberglass tapes and averaged. Skinfolds were measured thrice to the nearest 0.2 mm by using Holtain calipers (Holtain Ltd) and averaged. Pediatricians assessed newborns for congenital abnormalities.

Newborn anthropometric measures were missed for 32% of births either because women moved to their parents’ village for the delivery (23%) or (for Mumbai births) because of late notification (3%), the team was denied access to the hospital (2%), or the newborn was acutely unwell (3%). Gestational age was calculated from the last menstrual period date unless different by greater than ±14 d from that estimated by a <20-wk ultrasound scan (9%) when the latter was used.

### Outcomes

Primary birth outcomes were birth weight and rates of low birth weight. Secondary birth outcomes were gestational age, small for gestational age, other newborn body measurements, operative delivery rates, intrauterine deaths or stillbirths, major congenital malformations, and twin or triplet pregnancies.

### Losses to follow-up

If women stopped attending for supplementation, we monitored periods and pregnancies by home visits. With the recording of multiple mobile phone numbers for each family, including those of friends and neighbors, we were able to keep in touch with many families who moved out of the immediate study area but remained within Mumbai or its suburbs. Women were designated lost to follow-up if they declined additional contact or moved away and were untraceable >6 mo.

### Power

Before the start of the trial, we estimated that, on the basis of an SD for birth weight of 600 g in this population, a sample size of 1500 subjects (750 subjects/group) would give ≥85% power to detect an increase in birth weight in the treatment group of 100 g and an interaction between allocation group and maternal BMI (expected effect of 125 g in the lowest one-third, 100 g in the middle one-third, and 75g in the highest one-third) significant at the 5% level.

### Changes in protocol

Initially, we followed up pregnancies only if the women started supplementation ≥90 d before their last menstrual period. Women who became pregnant sooner than this were excluded from additional supplementation and follow-up. This was a pragmatic strategy to limit costs. However, the exclusion of women caused disappointment, and from December 2008, we decided to follow up all pregnancies and censor data at the analysis stage. As previously described, treatment snacks changed during the trial to finally incorporate 100% of fresh green leafy vegetables in response to women's comments on palatability, and the randomization method changed.

### Ethics

The trial (www.controlled-trials.com/isrctn/; ISRCTN62811278) was approved by the ethics committees of BYL Nair and TN Medical College, Grant Medical College, and Sir JJ Group of Hospitals, Mumbai, and Southampton and SW Local Research Ethics Committees. An independent data-monitoring committee reviewed data every 6 mo for 2 y and then annually. Stopping rules, which were based on adverse incidents (abortions, maternal deaths, preterm births, LBW, congenital abnormalities, stillbirths and infant deaths) were predetermined. The trial protocol can be obtained from the corresponding author.

### Statistics

We compared baseline measurements between allocation groups and between women who remained in the study or dropped out. We carried out an intention-to-treat analysis by comparing newborn measurements between allocation groups in all women who were randomly assigned and became pregnant after starting supplementation and a per-protocol analysis that was limited to women supplemented ≥90 d before their last menstrual period date. In both analyses, intrauterine deaths, stillbirths, twin or triplet pregnancies, and major congenital abnormalities were included only to compare their prevalence between groups and then excluded, and the analysis was limited to live singleton newborns measured within 10 d. We hypothesized, a priori, an interaction between the allocation group and maternal prepregnant BMI; in regression models, we used an interaction term (allocation group × BMI as a continuous variable), and for the presentation of interaction effects in tables and figures, we used categories (thirds) of maternal BMI (in kg/m^2^; <18.6, 18.6–21.8, and >21.8). We also tested for interactions with maternal age and height (continuous variables), parity (binary variable; 0 = primiparous, 1 = at least one previous delivery), and newborn sex (binary variable). Small-for-gestational-age and large-for-gestational-age births were defined according to Oken et al (21) and preterm as gestation <37 wk. Comparisons were made by using *t* tests, Mann-Whitney *U* tests, and chi-square or Fisher's exact tests for normally distributed continuous, nonparametric, and categorical variables, respectively. Primary results are reported unadjusted; we used multiple regression analysis to assess intervention effects with adjustment for gestational age, infant sex, age of newborn measurement, maternal BMI, height, parity, age, socioeconomic status, gestational diabetes, education, compliance, and baseline food intakes. Finally, we examined the intervention effect on birth weight at different levels of maternal compliance. Results were considered statistically significant at *P* < 0.05. The analysis was performed with STATA software (version 12.1; StataCorp LP).

## RESULTS

**FIGURE 1.**
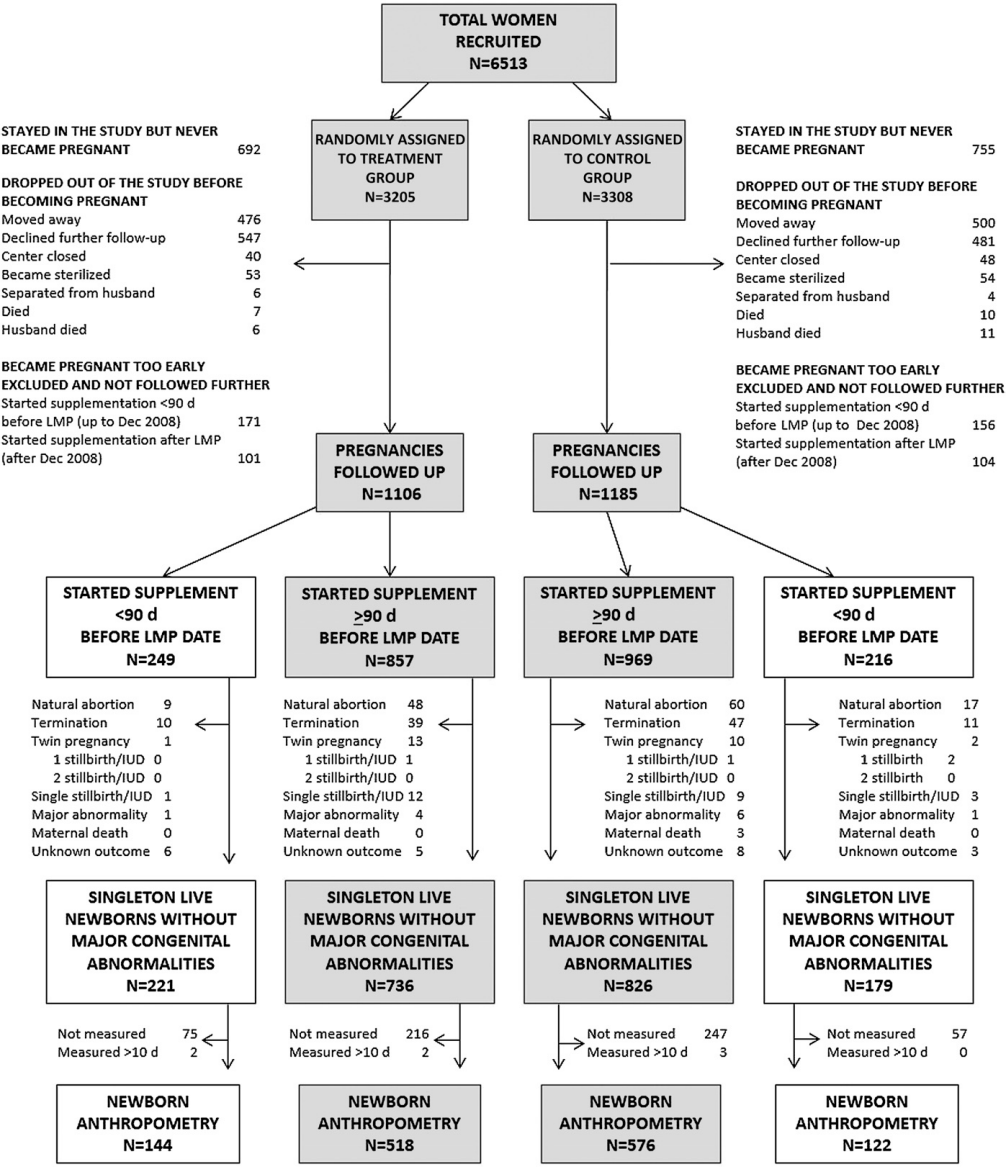
Consolidated Standards of Reporting Trials diagram showing participant flow in the trial. To make all figures mutually exclusive, if a major congenital abnormality was detected on a scan and led to an abortion or termination, this case was classified as a major congenital abnormality and did not appear under abortion or termination. Shaded boxes indicate women who started supplementation ≥90 d before their LMP. IUD, intrauterine fetal death; LMP, last menstrual period date.

A total of 6513 women were eligible and participated (**Figure 1**). Baseline characteristics were similar in the 2 allocation groups (**Table 3**). At recruitment, 32% of women were underweight (BMI <18.5), and 14% of women were overweight or obese (BMI >25). One-third of women were nulliparous. Only 6 women smoked, and 10% of women chewed tobacco. Eighty-seven percent of women had completed at least a secondary education. Only 21% of women were in paid employment, mainly unskilled or semiskilled. Food-frequency questionnaire data collected at enrollment showed that women's diets were monotonous, with low intakes of micronutrient-rich foods. One-half of the women had not consumed milk or milk products (eg, yogurt) in the preceding week other than in tea, and one-quarter of women had not consumed any green leafy vegetables. The majority of women (85%) had eaten fruit <1 time/d, and more than one-quarter of women had consumed no meat or fish in the preceding week.

**TABLE 3:** Baseline characteristics of women in the treatment and control groups (all 6513 women enrolled)

	Treatment (*n* = 3205)		Control (*n* = 3308)	
	*n*	Value	*n*	Value
Age (y)	3205	25 (22, 28)*^1^*	3308	25 (22, 28)
Weight (kg)	3204	45.8 (40.4, 53.0)	3308	46.2 (40.5, 53.0)
Height (cm)	3203	151.3 ± 5.5*^2^*	3305	151.2 ± 5.5
BMI (kg/m^2^)	3202	20.0 (17.9, 23.0)	3305	20.1 (17.9, 22.9)
Parity*^3^* [*n* (%)]	3204	—	3308	—
0	—	1003 (31.3)	—	996 (30.1)
1	—	1399 (43.7)	—	1464 (44.3)
>1	—	802 (25.0)	—	848 (25.6)
Tobacco user*^3^* [*n* (%)]	3205	315 (9.8)	3308	345 (10.4)
Standard of living index	3027	24.4 ± 6.1	3130	24.5 ± 6.1
Religion*^3^* [*n* (%)]	3205	—	3303	—
Hindu	—	2233 (69.7)	—	2328 (70.5)
Muslim	—	822 (25.6)	—	849 (25.7)
Other	—	150 (4.7)	—	126 (3.8)
Education*^3^* [*n* (%)]	3199	—	3305	—
Primary or less	—	413 (12.9)		396 (12.0)
Secondary	—	2604 (81.4)		2735 (82.8)
Graduate	—	182 (5.7)		174 (5.3)
Occupation*^3^* [*n* (%)]	3205		3308	—
Semiskilled/unskilled	—	530 (16.5)	—	572 (17.3)
Skilled/self-employed	—	85 (2.7)	—	95 (2.9)
Professional	—	52 (1.6)	—	64 (1.9)
Not working	—	2538 (79.2)	—	2577 (77.9)
Husband's education*^3^* [*n* (%)]	3178	—	3288	—
Primary or less	—	235 (7.4)	—	224 (6.8)
Secondary	—	2676 (84.2)	—	2791 (84.9)
Graduate	—	267 (8.4)	—	273 (8.3)
Husband's occupation*^3^* [*n* (%)]	3205	—	3308	—
Semi-skilled/unskilled	—	1997 (62.3)		2013 (60.9)
Skilled/self-employed	—	925 (28.9)		1012 (30.6)
Professional	—	204 (6.4)		209 (6.3)
Not working/other	—	79 (2.5)		74 (2.2)
First language*^3^* [*n* (%)]	3203	—	3301	—
Marathi	—	1651 (51.5)	—	1693 (51.3)
Hindi	—	1217 (38.0)	—	1240 (37.6)
Other	—	335 (10.5)	—	368 (11.1)
Dietary intake*^3^* [*n* (%)]	3205	—	3308	—
Milk and milk products (other than in tea)	—	—	—	
<1 time/wk	—	1569 (49.0)	—	1630 (49.3)
1–6 times/wk	—	1175 (36.7)	—	1233 (37.3)
≥7 times/wk	—	461 (14.4)	—	445 (13.5)
GLVs*^4^*	—	—	—	—
<1 time/wk	—	750 (23.4)	—	807 (24.4)
1–6 times/wk	—	2359 (73.6)	—	2408 (72.8)
≥7 times/wk	—	96 (3.0)	—	93 (2.8)
Fruit	—	—	—	—
<1 time/wk	—	537 (16.8)	—	585 (17.7)
1–6 times/wk	—	2151 (67.1)	—	2217 (67.0)
≥7 times/wk	—	517 (16.1)	—	506 (15.3)
Meat and fish	—	—	—	—
<1 time/wk	—	853 (26.6)	—	882 (26.7)
1–6 times/wk	—	2024 (63.2)	—	2107 (63.7)
≥7 times/wk	—	328 (10.2)	—	319 (9.6)

1 Median; IQR in parentheses (all such values).

2 Mean ± SD (all such values for normally distributed variables).

3 Categorical variable.

4 GLV, green leafy vegetable.

A total of 1447 women (treatment: 22%; control: 23%) stayed in the trial throughout but never became pregnant (Figure 1). An additional 2243 women (treatment: 35%; control: 34%) dropped out before becoming pregnant either because they moved away (usually because of slum redevelopment), declined additional follow-up, died, separated from their husbands, or were sterilized. Women who stayed in the study were better educated, of higher socioeconomic status, and had lower meat and fish intakes than did women who dropped out but did not differ between allocation groups (*see* Supplemental Table 2 under “Supplemental data” in the online issue). We excluded 532 women (8% in both groups) who became pregnant too early (before December 2008: <90 d after starting supplementation; after December 2008: before starting supplementation). The remaining 2291 women were followed up through pregnancy.

### Intention-to-treat analysis

The 2291 pregnancies resulted in 1962 live singleton newborns without major congenital abnormalities; 1360 of these newborns were measured.

#### Newborn measurements

**FIGURE 2.**
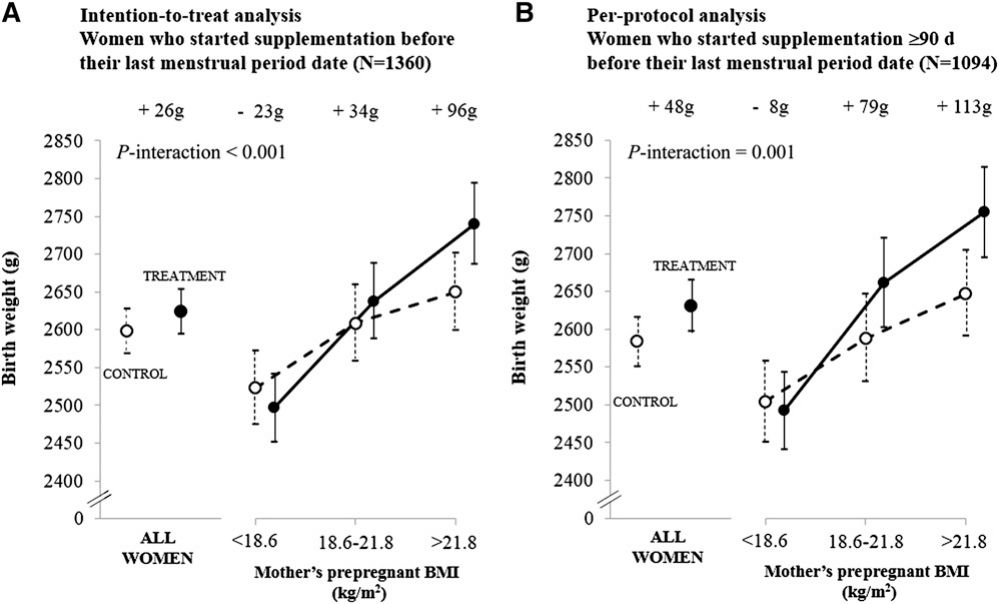
Effect of the intervention on birth weight according to categories of maternal prepregnant BMI: intention-to-treat analysis (A) and per-protocol analysis (B). Values are means; error bars indicate 95% CIs. *P*-interaction values between the allocation group (0, 1) and maternal prepregnant BMI (continuous variable) were derived by using a product term (allocation group × BMI) in linear regression models.

**FIGURE 3.**
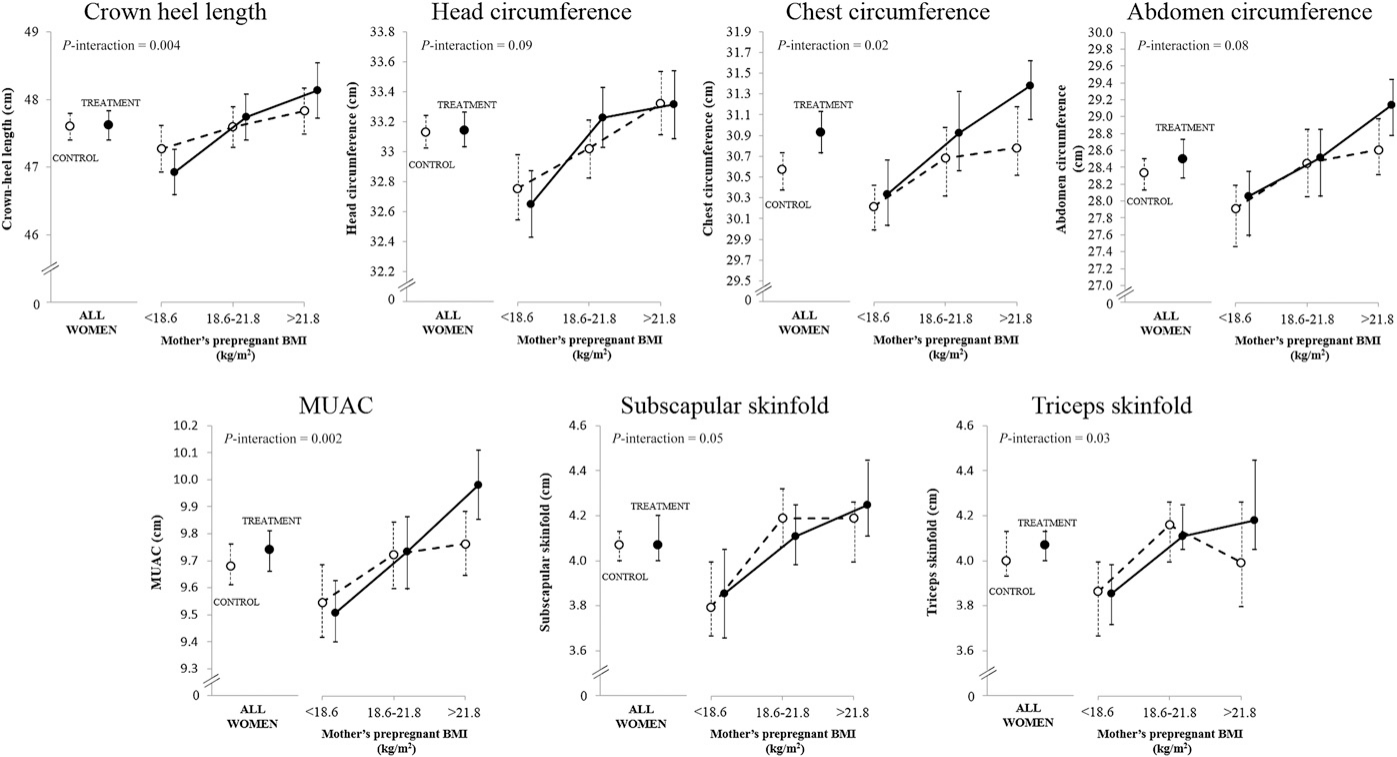
Effect of the intervention on other birth measurements according to categories of maternal BMI (per-protocol analysis; women who started supplementation ≥90 d before their last menstrual period date). Values are means; error bars indicate 95% CIs. *P*-interaction values between the allocation group (0, 1) and maternal prepregnant BMI (continuous variable) were derived by using product terms (allocation group × BMI) in linear regression models. MUAC, midupper arm circumference.

In unadjusted analyses, the median (IQR) gestational age was 39.0 wk (37.9–40.0 wk) and 39.1 wk (38.0–40.0 wk) in treatment and control groups, respectively (*P*-difference = 0.50). There were no significant differences between allocation groups in birth weight (treatment: 2624 g; control: 2598 g; *P* = 0.22; +26 g; 95% CI: −15, 68 g); **Figure 2**], percentages of LBW (treatment: 34%; control: 39%; OR: 0.83; 95% CI: 0.66, 1.05; *P* = 0.10), small-for-gestational age-births (treatment: 67%; control: 69%, OR: 0.89; 95% CI: 0.70, 1.13; *P* = 0.33), large-for-gestational-age births (treatment: 0.5%; control: 0.4%; *P* = 1.0), or preterm births (treatment: 13%; control: 12%; *P* = 0.60). There were interactions between the allocation group and maternal prepregnant BMI for birth weight (*P*-interaction < 0.001) (Figure 2) and other newborn measurements (**Figure 3**; *see* Supplemental Table 3 under “Supplemental data” in the online issue) such that the intervention effect was greater in mothers of higher BMI. Percentages of LBW infants in thirds of maternal BMI were as follows: maternal BMI <18.6: treatment: 44%; control: 44% (OR: 0.99; 95% CI: 0.68, 1.45); maternal BMI from 18.6 to 21.8: treatment: 32%; control: 39% (OR: 0.74; 95% CI: 0.49, 1.10); maternal BMI >21.8: treatment: 25%; control: 32% (OR: 0.69; 95% CI: 0.44, 1.08) (*P*-interaction = 0.008). Results were similar for gestation-adjusted birth measurements (not shown) and in the regression analysis adjusted for other factors influencing birth size (**Table 4**).

**TABLE 4:** Multiple linear regression analysis for the effect on birth weight in the intention-to-treat and per-protocol analyses*^1^*

	Intention-to-treat analysis (women who started supplementation before their LMP date)		Per-protocol analysis (women who started supplementation ≥90 d before their LMP date)	
	Effect on birth weight (g)	*P*	Effect on birth weight (g)	*P*
Effect of intervention				
If maternal BMI <18.6 kg/m^2^	28.5 (−46.8, 103.8)	0.458	26.3 (−57.7, 110.3)	0.539
If maternal BMI from 18.6 to 21.8 kg/m^2^	68.1 (−8.1, 144.4)	0.080	86.0 (−0.5, 172.4)	0.051
If maternal BMI >21.8 kg/m^2^	148.2 (70.4, 226.0)	<0.001	133.8 (46.0, 221.6)	0.003
Maternal BMI				
<18.6	Ref	Ref	Ref	Ref
18.6–21.8	84.7 (19.5, 149.9)	0.011	90.3 (18.0, 162.7)	0.014
>21.8	86.8 (19.5, 154.2)	0.012	105.2 (29.6, 180.7)	0.006
Maternal				
Height (cm)	11.4 (7.8, 14.9)	<0.001	13.3 (9.3, 17.3)	<0.001
Age (y)	−1.8 (−7.4, 3.8)	0.519	−1.4 (−7.8, 5.0)	0.665
Standard of Living Index (score)*^2^*	2.8 (−0.7, 6.2)	0.113	1.2 (−2.5, 5.0)	0.525
Gestational diabetes	−15.7 (−101.0, 69.7)	0.719	14.1 (−79.9, 108.2)	0.768
Missing GTT (intention to treat: *n* = 572; per protocol: *n* = 469)	−1.3 (−41.9, 39.3)	0.951	1.7 (−43.9, 47.4)	0.941
Parity				
0	Ref	Ref	Ref	Ref
1	114.2 (67.4, 161.0)	<0.001	117.5 (62.8, 172.3)	<0.001
>1	156.3 (96.3, 216.3)	<0.001	147.4 (78.9, 215.9)	<0.001
Education				
Primary or less	Ref	Ref	Ref	Ref
Secondary	12.3 (−57.0, 81.5)	0.728	−1.7 (−80.4, 77.1)	0.967
Graduate	65.2 (−43.4, 173.8)	0.239	33.5 (−89.6, 156.6)	0.594
Noncompliant*^3^*	Ref	Ref	Ref	Ref
Compliant*^3^*	21.2 (−33.4, 75.8)	0.446	−6.2 (−66.8, 54.4)	0.841
Compliance × allocation group (control: 0; treatment: 1)	−88.3 (−165.9, −10.8)	0.026	−46.1 (−133.6, 41.4)	0.301
Prepregnant frequency (±SD) of intake per week*^4^*				
Milk	29.5 (5.7, 53.3)	0.015	30.3 (3.4, 57.1)	0.027
Green leafy vegetables	−24.2 (−45.5, −3.0)	0.026	−26.9 (−51.1, −2.8)	0.029
Fruit	11.4 (−9.9, 32.7)	0.294	7.1 (−17.0, 31.2)	0.565
Newborn				
Sex (girl: 0; boy: 1)	110.3 (71.8, 148.9)	<0.001	111.7 (68.2, 155.3	<0.001
Gestational age (wk)	63.0 (52.7, 73.2)	<0.001	67.2 (55.5, 78.8	<0.001
Age when measured*^5^*				
0 d [(a) *n* = 169; (b) *n* = 134]	Ref	Ref	Ref	Ref
1–3 d [(a) *n* = 831; (b) *n* = 660]	−76.1 (−134.8, −17.5)	0.011	−81.0 (−147.0, −15.0)	0.016
>3 d [(a) *n* = 294; (b) *n* = 231]	−9.1 (−76.9, 58.8)	0.793	−0.1 (−76.5, 76.3)	0.999
Intercept	−1668.6 (−2346.7, −990.4)	<0.001	−2102.3 (−2871.2, −1333.5)	<0.001

1 All values are regression coefficients; 95% CIs in parentheses. All variables shown were included in the model together on the basis of 1294 (intention to treat) and 1025 (per protocol) pregnancies with complete data for all variables except GTT data. GTT, glucose tolerance test; LMP, last menstrual period; Ref, reference group.

2 Indicator of socioeconomic status (*see* Recruitment and baseline investigations in Subjects and Methods).

3 Compliance: categorical variable was 1 if the total number of supplements consumed in the 90 d before the LMP date up to delivery divided by the total number it was possible to have eaten in that time was ≥0.5; otherwise, the categorical variable was 0.

4 Variables were Fisher-Yates transformed (22).

5 Intention-to-treat analysis group indicated by (a). Per-protocol analysis group indicated by (b).

#### Other outcomes

There were more male than female newborns (54% compared with 46% in both allocation groups). Elective cesarean delivery, emergency cesarean delivery, and forceps/ventouse rates did not differ between intervention groups (treatment: 9.5%, 11.9% and 0.6%, respectively; control: 10.2%, 11.2%, and 1.0%, respectively). There were 25 singleton intrauterine deaths or stillbirths (Figure 1; treatment: 1.2% of pregnancies; control: 1.0% of pregnancies); 12 major congenital abnormalities (major heart defects, neural tube defects, skeletal dysplasias, and Down syndrome; treatment: 0.5%; control: 0.6%); and 26 twin and triplet pregnancies (treatment: 1.3%; control: 1.0%). Numbers for all outcomes were similar in both allocation groups.

### Per-protocol analysis

Of 2291 pregnant women, 1826 women started supplementation ≥90 d before their last menstrual periods (Figure 1, shaded boxes). These women delivered 1562 live singleton newborns without major congenital abnormalities; 1094 of these newborns were measured.

#### Newborn measurements

In unadjusted analyses, the median (IQR) gestation was 39.1 wk (38.0–40.0 wk) in both allocation groups. Birth weight was higher in the treatment group by 48 g (95% CI: 1, 96 g; *P* = 0.046) (Figure 2). Percentages of LBW and small-for-gestational-age births were lower [LBW: treatment: 34%; control: 41%; OR: 0.76; 95% CI: 0.59, 0.98 (*P* = 0.03); small-for-gestational-age: treatment: 66%; control: 71%; OR: 0.80; 95% CI: 0.61, 1.04 (*P* = 0.09)]. Percentages of large-for-gestational-age infants (treatment: 0.6%; control: 0.5%; *P* = 1.0) and preterms (treatment: 12.7%; control: 12.3%; *P* = 0.87) were similar in both groups. As in the larger group of women, there were interactions between the allocation group and maternal prepregnant BMI for birth weight (*P*-interaction = 0.001) (Figure 2) and other newborn measurements (Figure 3; *see* Supplemental Table 3 under “Supplemental data” in the online issue). In the highest one-third of maternal BMI, birth weight increased by 113 g (95% CI: 29, 197 g), birth length increased by 0.3 cm (95% CI: −0.2, 0.9 cm), chest circumference increased by 0.6 cm (95% CI: 0.2, 1.1 cm), midupper arm circumference increased by 0.2 cm (95% CI: 0.1, 0.4 cm), and triceps skinfold thickness increased by 0.2 mm (95% CI: −0.1, 0.5 mm). Percentages of LBW infants in thirds of maternal BMI were as follows—lowest: treatment: 45%; control: 46% (OR: 0.95; 95% CI: 0.62, 1.44); middle: treatment: 31%; control: 42% (OR: 0.63; 95% CI: 0.40, 0.99); and highest: treatment: 24%; control: 34% (OR: 0.61; 95% CI: 0.36, 1.01) (*P*-interaction = 0.01). Results were similar for gestation-adjusted birth measurements (not shown) and in the regression analysis (Table 4).

#### Other outcomes

Elective and emergency cesarean delivery and forceps/ventouse rates were similar in both allocation groups (treatment: 9.6%, 11.3%, and 0.6%, respectively; control: 10.1%, 10.7%, and 0.9%, respectively). There were 21 singleton intrauterine deaths or stillbirths (Figure 1; treatment: 1.4%; control: 0.9%), 10 major congenital abnormalities (treatment: 0.5%; control: 0.6%), and 23 twin and triplet pregnancies (treatment: 1.5%; control: 1.0%). Numbers for all other outcomes were similar in both allocation groups.

There were no consistent interactions between the allocation group and maternal age, parity, height, or newborn sex in relation to any of the outcomes in either the intention-to-treat or per-protocol analyses.

### Compliance

Fewer women in the treatment group than control group were compliant (45% compared with 57%, respectively). Compliance fell in the May through June holiday season each year and during major festivals. Compliance was unrelated to maternal BMI or socioeconomic status and was similar in the 3 mo before pregnancy (treatment: 44%; control: 53%) and during pregnancy (treatment: 38%; control: 49%). Individual compliance fell with an increasing length of time in the study. There was no evidence of a larger intervention effect in compliant women, and indeed, compliance was associated with a reduction in the effect of the intervention on birth weight in the intention-to-treat analysis (Table 4; *see* Supplemental Table 4 and Supplemental Figure 1 under “Supplemental data” in the online issue). This result remained true after adjustment for additional potential confounders (household size, religion, tobacco use, supplementation center, timing of the pregnancy within the trial, and length of time in the study).

## DISCUSSION

This randomized controlled trial used the following 2 novel approaches: supplementing mothers with local micronutrient-rich foods to improve diet quality and starting before conception to increase birth weight. In the intention-to-treat analysis (all pregnancies), there was no overall effect on birth weight or the prevalence of LBW. In women who started supplementation ≥3 mo before pregnancy (per-protocol analysis), there was a mean 48-g increase in birth weight and a reduction in LBW of 24%. In both analyses, the intervention effect was conditioned by maternal BMI in a direction opposite to that initially hypothesized. There was no apparent effect in underweight mothers and an effect of +63 g (95% CI: 11, 115 g; intention-to-treat analysis) and +94 g (95% CI: 35, 154 g; per-protocol analysis) in mothers with BMI ≥18.6. There were similar effects on other birth measurements (secondary outcomes), which were strongest for arm and chest circumferences. There were no evident effects on gestational age or (though numbers were small) multiple pregnancies, intrauterine deaths and stillbirths, operative deliveries, or major congenital abnormalities.

### Strengths and limitations

Strengths of the study were the individual random assignment, supervised supplementation, menstrual period monitoring combined with ultrasound to date pregnancies, and standardized measurements of glucose tolerance during pregnancy. The study was carried out in a slum population at high risk of LBW. The use of health workers from the community maximized participant cooperation. A limitation was that the slum community was quite mobile, which led to a loss to follow-up of 34%. Women who stayed in the study differed from those who dropped out (s*ee* Supplemental Table 2 under “Supplemental data” in the online issue). However, differences were small, comparable in both allocation groups, and unlikely to generate spurious effects or compromise the generalizability. Full blinding was impossible, and if women thought they were getting the healthier snack, this belief could have modified their behaviors in other ways favoring a better pregnancy outcome. However, this effect would not explain the BMI interaction. We did not monitor women's compliance with routine iron and folic acid supplements, which are known to increase birth weight (23). Several factors could have attenuated the intervention effect. Thirty-two percent of newborns were not measured, which reduced the sample size. Only 40–50% of women were fully compliant, and compliance was lower in the treatment group than control group. Pregnancy outcomes may have improved in both groups; both groups received antenatal monitoring and encouragement to take iron plus folate supplements. We had no baseline population-level birth-weight data in this community to assess this possibility. Control women may have increased their habitual intakes of green leafy vegetables, although such an increase was not supported by serial food-frequency questionnaire data (not shown). Although our ultrasonologist did not divulge the sex of the fetus, the high percentage of male births suggested that some parents obtained this information from private ultrasound clinics and opted for termination if the fetus was female, which is a well-recognized but illegal practice in India (24).

### Interaction with maternal BMI

Our supplement appeared to have larger effects on the newborn size in mothers of higher BMI. This effect was the opposite of what we hypothesized on the basis of observational data available when we designed the trial (9).Therefore, this result could have been a chance finding and should be interpreted with caution. The result was opposite to the effect seen in trials of protein-energy supplementation in pregnancy, which increases birth weight more in undernourished women (25). However, the result was consistent with data from multiple micronutrient trials in pregnancy; a meta-analysis of individual-level data from 12 trials showed an interaction between the allocation group and maternal BMI with a greater birth-weight effect in mothers of higher BMI in 11 of the trials (4). We speculate that underweight mothers in our study may have had inadequate macronutrients or other substrates to use nutrients supplied by our supplements or partition them to the fetus. A nutritional intervention will only have a benefit up to the point at which other nutrient deficiencies become limiting. The metabolism of nutrients, development of the fetal supply line, transportation of nutrients across the placenta, and fetal growth require energy and other substrates. Our interpretation of the BMI interaction is that extra macronutrients, in addition to micronutrients, may be needed for underweight women in low- and middle-income countries; a trial in Burkina Faso of energy (1.6 MJ) and protein (14.7 g) plus multiple micronutrients in pregnancy showed a greater effect on birth size than with the use of micronutrients alone and greater effects in underweight women (26).

### Source of the effect

If the effect of the supplement on birth weight was real, it may have resulted from micronutrients in the snacks or other important compounds (eg, fatty acids) in foods. Observational studies in high-income populations have linked higher maternal intakes of cow milk with higher birth weight (27). To our knowledge, there are no equivalent data for green leafy vegetables or fruit. The effect may have resulted from the higher energy (+0.32 MJ) and/or protein (+4 g) in treatment than control snacks. However, trials that showed an effect of protein and energy supplementation on birth weight used more (2–10 times) energy and protein than were present in our treatment snacks (25).

### Timing of intervention

Although the birth-weight effect was slightly larger in women who started supplementation ≥3 mo before becoming pregnant, our results suggested that the intervention had similar efficacy irrespective of the duration of supplementation preconceptionally. The trial was not designed to answer another important question of whether preconceptional supplementation is more effective than starting supplementation, as is more usual, after pregnancy has been diagnosed. There has been a paucity of data on short- and long-term effects of preconceptional supplementation in humans.

### Compliance

We did not find larger effects on birth weight in women who ate more supplements; indeed, the intention-to-treat analysis showed a smaller effect on birth weight in fully compliant women. We are unable to explain this result. The finding could reflect confounding; the most-deprived women may have been hungrier and, therefore, more compliant and also had smaller infants. However, adjustment for multiple confounders did not alter the effect. There may have been contaminants (eg, pesticides) in the green leafy vegetables, which became toxic at higher intakes, but we washed the fresh leaves thoroughly. We speculate that, because women were most compliant in the first weeks and months of supplementation, women who were in the study for longer may have benefited from supplementation over a longer period even if their compliance was lower immediately before and during pregnancy (the period used to define compliance).

In conclusion, the findings from this trial clearly do not have any immediate implications for policies to improve maternal nutritional status and prevent LBW. The effect on birth weight was significant only in the per-protocol analysis and would need to be replicated to have confidence that it is real. The effect was modest but similar in magnitude to that achieved by using multiple micronutrient supplements in pregnancy, which suggests that food-based approaches may have a role. The effect was apparently present only in mothers who were not underweight. This finding suggests that women of different nutritional status may need different interventions, which could be extremely challenging in a programmatic setting. However, it is a potentially important finding which, if replicated, needs to be understood biologically. We are following up the children born during the trial to assess whether this intervention, which covered the periconceptional period (and, therefore, epigenetic changes) and the first trimester (and, therefore, organogenesis), has any longer-term functional and health effects in the offspring. The results of these studies will determine whether this intervention is worth pursuing further.

## Supplementary Material

Supplemental data
