# Supplementary material for: Improving women's diet quality preconceptionally and during gestation: effects on birth weight and prevalence of low birth weight—a randomized controlled efficacy trial in India (Mumbai Maternal Nutrition Project)1
Source: Am J Clin Nutr. 2014 Sep 17;100(5):1257–68. doi: 10.3945/ajcn.114.084921 (PMC4196482; doi:10.3945/ajcn.114.084921)
Supplement: Supplemental data [file supp_100_5_1257__index.html]

Supplemental data 

# Improving women's diet quality preconceptionally and during gestation: effects on birth weight and prevalence of low birth weight—a randomized controlled efficacy trial in India (Mumbai Maternal Nutrition Project)

## Supplemental data

**Files in this Data Supplement:**

- Supplemental data - Figure 1
- Supplemental data - Tables 1-4 and Figure Legend
