# Supplementary figures and images for: Improving women's diet quality preconceptionally and during gestation: effects on birth weight and prevalence of low birth weight—a randomized controlled efficacy trial in India (Mumbai Maternal Nutrition Project)1
Source: Am J Clin Nutr. 2014 Sep 17;100(5):1257–68. doi: 10.3945/ajcn.114.084921 (PMC4196482; doi:10.3945/ajcn.114.084921)

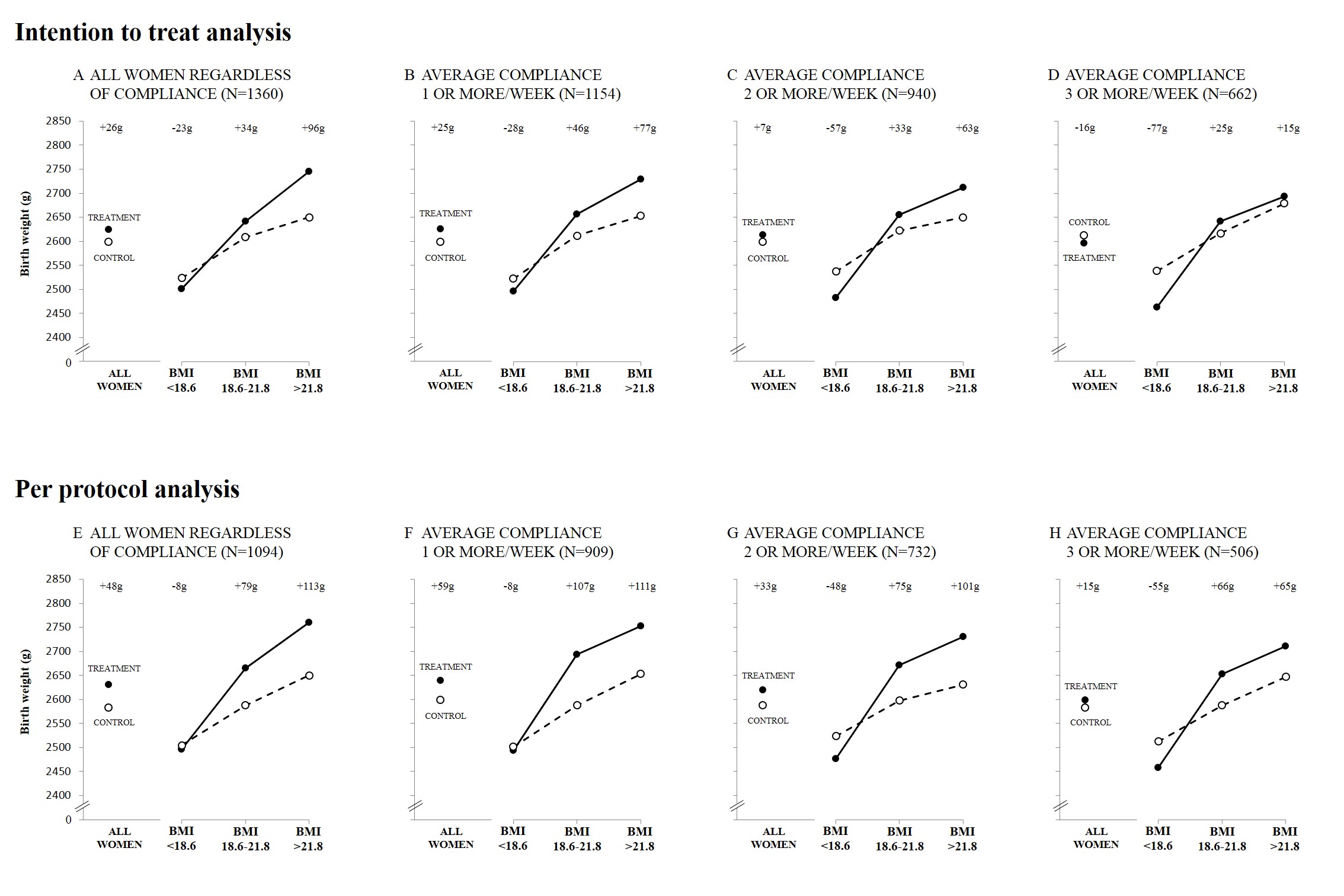

Supplement: Supplemental data [file 114.084921_ajcn084921SupplementaryData1.jpg]
