# Supplementary material for: Improving women's diet quality preconceptionally and during gestation: effects on birth weight and prevalence of low birth weight—a randomized controlled efficacy trial in India (Mumbai Maternal Nutrition Project)1
Source: Am J Clin Nutr. 2014 Sep 17;100(5):1257–68. doi: 10.3945/ajcn.114.084921 (PMC4196482; doi:10.3945/ajcn.114.084921)
Supplement: Supplemental data [file 114.084921_ajcn084921SupplementaryData2.doc]

**SUPPLEMENTAL TABLES and SUPPLEMENTAL FIGURE LEGEND**

**Supplemental Table 1: Examples of Treatment and Control Recipes.**

**Treatment** Recipes Control Recipes

| **Ingredient** | **Weight** |  | **Ingredient** | **Weight (g)** |
| --- | --- | --- | --- | --- |
| *Recipe 1* |  |  | *Recipe 1* |  |
| Fresh spring onion stalk | 15 |  | Tapioca | 30 |
| Whole milk powder | 12 |  | Potato | 10 |
| Dried raisins and figs | 4 |  | Corn flour | 2.5 |
| Wheat flour | 7 |  | Wheat flour | 2.5 |
| Rice flour | 6 |  | Mixed spices | 1 |
| Sorghum flour | 0.5 |  |  |  |
| Pearl millet flour | 4 |  | *Recipe 2* |  |
| Mixed spices | 1 |  | Potato | 45 |
|  |  |  | Wheat flour | 5 |
| *Recipe 2* |  |  | Corn flour | 2 |
| Fresh onion stalk | 10 |  | Mixed spices | 1 |
| Fresh coriander | 10 |  |  |  |
| Whole milk powder | 12 |  | *Recipe 3* |  |
| Dried apricot | 4 |  | Potato | 25 |
| Wheat flour | 19 |  | Semolina | 5 |
| Pearl millet flour | 5 |  | Rice flour | 15 |
| Mixed spices | 1 |  | Mixed spices | 1 |
|  |  |  |  |  |
| *Recipe 3 (fruit bar)* |  |  | *Recipe 4 (‘chikki’)* |  |
| Mango | 30 |  | Puffed rice | 7 |
| Indian gooseberry | 17 |  | Jaggery | 2 |
| Raisins | 5 |  | Sugar | 4 |
| Chickpea | 10 |  |  |  |
| Sesame seed | 3 |  |  |  |

All treatment and control snacks except the fruit bar and chikki were cooked by deep-frying in sunflower oil.

**Supplemental Table 2: Comparison of baseline characteristics between women who dropped out of the study and women who remained^1^**, and further sub-divided according to allocation group

|  | **N** | **REMAINED IN THE STUDY**  **N = 3738** | |  | **N** | **DROPPED OUT OF THE STUDY**  **N = 2243** | | **p** |  | **REMAINED IN THE STUDY** | | | |  | **DROPPED OUT OF THE STUDY** | | | |
| --- | --- | --- | --- | --- | --- | --- | --- | --- | --- | --- | --- | --- | --- | --- | --- | --- | --- | --- |
|  |  |  |  |  |  |  |  |  |  | **TREATMENT**  **N=1798** | | **CONTROL**  **N=1940** | |  | **TREATMENT**  **N=1135** | | **CONTROL**  **N=1108** | |
|  |  | **Median** | (IQR) |  |  | **Median** | (IQR) |  |  | **Median** | (IQR) | **Median** | (IQR) |  | **Median** | (IQR) | **Median** | (IQR) |
|  |  |  |  |  |  |  |  |  |  |  |  |  |  |  |  |  |  |  |
| Age (years) | **3738** | **25.0** | (22.0, 28.0) |  | **2243** | **25.0** | (22.0, 28.0) | 0.03^4^ |  | **25.0** | (22.0, 28.0) | **25.0** | (22.0, 28.0) |  | **25.0** | (22.0, 28.0) | **25.0** | (22.0, 28.0) |
| Weight (kg) | **3737** | **46.1** | (40.5, 52.8) |  | **2243** | **46.2** | (40.5, 53.4) | 0.58^†^ |  | **45.9** | (40.3, 52.8) | **46.3** | (40.7, 52.7) |  | **46.0** | (40.6, 53.4) | **46.3** | (40.3, 53.5) |
| Height (cm)^2^ | **3736** | **151.4** | (5.5) |  | **2240** | **151.1** | (5.5) | 0.06 |  | **151.3** | (5.5) | **151.4** | (5.5) |  | **151.2** | (5.4) | **150.9** | (5.5) |
| Body mass index (kg/m^2^) | **3735** | **20.0** | (18.0, 22.8) |  | **2240** | **20.2** | (17.9, 23.2) | 0.16^4^ |  | **20.0** | (17.9, 22.9) | **20.1** | (18.0, 22.8) |  | **20.0** | (17.9, 23.3) | **20.5** | (17.8, 23.2) |
| Parity^3^ 0 | **3737** | **1127** | (30.2) |  | **2243** | **627** | (28.0) | <0.001^5^ |  | **568** | (31.6) | **559** | (28.8) |  | **315** | (27.8) | **312** | (28.2) |
| 1 |  | **1749** | (46.8) |  |  | **930** | (41.5) |  |  | **835** | (46.5) | **914** | (47.1) |  | **467** | (41.1) | **463** | (41.8) |
| >1 |  | **861** | (23.0) |  |  | **686** | (30.6) |  |  | **394** | (21.9) | **467** | (24.1) |  | **353** | (31.1) | **333** | (30.1) |
| Tobacco user^3^ | **3738** | **351** | (9.4) |  | **2243** | **256** | (11.4) | 0.01^5^ |  | **166** | (9.2) | **185** | (9.5) |  | **125** | (11.0) | **131** | (11.8) |
|  |  |  |  |  |  |  |  |  |  |  |  |  |  |  |  |  |  |  |
| Standard of living index^2^ | **3647** | **25.0** | (6.0) |  | **2024** | **23.6** | (6.2) | <0.001 |  | **25.0** | (5.9) | **25.0** | (6.0) |  | **23.7** | (6.2) | **23.6** | (6.3) |
| Religion^3^ Hindu | **3737** | **2690** | (72.0) |  | **2239** | **1514** | (67.6) | 0.002^5^ |  | **1297** | (72.1) | **1393** | (71.8) |  | **757** | (66.7) | **757** | (68.6) |
| Muslim |  | **899** | (24.1) |  |  | **625** | (27.9) |  |  | **429** | (23.9) | **470** | (24.2) |  | **316** | (27.8) | **309** | (28.0) |
| Other |  | **148** | (4.0) |  |  | **100** | (4.5) |  |  | **72** | (4.0) | **76** | (3.9) |  | **62** | (5.5) | **38** | (3.4) |
|  |  |  |  |  |  |  |  |  |  |  |  |  |  |  |  |  |  |  |
| Education^3^ |  |  |  |  |  |  |  |  |  |  |  |  |  |  |  |  |  |  |
| Primary or less | **3732** | **376** | (10.1) |  | **2242** | **359** | (16.0) | <0.001^5^ |  | **192** | (10.7) | **184** | (9.5) |  | **182** | (16.0) | **177** | (16.0) |
| Secondary |  | **3133** | (83.9) |  |  | **1775** | (79.2) |  |  | **1493** | (83.2) | **1640** | (84.6) |  | **893** | (78.7) | **882** | (79.6) |
| Graduate |  | **223** | (6.0) |  |  | **108** | (4.8) |  |  | **109** | (6.1) | **114** | (5.9) |  | **59** | (5.2) | **49** | (4.4) |
|  |  |  |  |  |  |  |  |  |  |  |  |  |  |  |  |  |  |  |
| Occupation^3^ |  |  |  |  |  |  |  |  |  |  |  |  |  |  |  |  |  |  |
| Semi-skilled/Unskilled | **3738** | **616** | (16.5) |  | **2243** | **395** | (17.6) | 0.10^5^ |  | **300** | (16.7) | **316** | (16.3) |  | **180** | (15.9) | **215** | (19.4) |
| Skilled/self-employed |  | **93** | (2.5) |  |  | **74** | (3.3) |  |  | **38** | (2.1) | **55** | (2.8) |  | **42** | (3.7) | **32** | (2.9) |
| Professional |  | **79** | (2.1) |  |  | **30** | (1.3) |  |  | **32** | (1.8) | **47** | (2.4) |  | **17** | (1.5) | **13** | (1.2) |
| Not working/Other |  | **2950** | (78.9) |  |  | **1744** | (77.8) |  |  | **1428** | (79.4) | **1522** | (78.5) |  | **896** | (78.9) | **848** | (76.5) |
|  |  |  |  |  |  |  |  |  |  |  |  |  |  |  |  |  |  |  |
| Husband’s education^3^ |  |  |  |  |  |  |  |  |  |  |  |  |  |  |  |  |  |  |
| Primary or less | **3721** | **233** | (6.3) |  | **2220** | **177** | (8.0) | <0.001^5^ |  | **124** | (6.9) | **109** | (5.6) |  | **89** | (7.9) | **88** | (8.0) |
| Secondary |  | **3159** | (84.9) |  |  | **1867** | (84.1) |  |  | **1509** | (84.3) | **1650** | (85.4) |  | **940** | (83.9) | **927** | (84.3) |
| Graduate |  | **329** | (8.8) |  |  | **176** | (7.9) |  |  | **156** | (8.7) | **173** | (9.0) |  | **92** | (8.2) | **84** | (7.6) |
|  |  |  |  |  |  |  |  |  |  |  |  |  |  |  |  |  |  |  |
| Husband’s occupation^3^ |  |  |  |  |  |  |  |  |  |  |  |  |  |  |  |  |  |  |
| Semi-skilled/Unskilled | **3738** | **2336** | (62.5) |  | **2243** | **1330** | (59.3) | <0.001^5^ |  | **1149** | (63.9) | **1187** | (61.2) |  | **671** | (59.1) | **659** | (59.5) |
| Skilled/self-employed |  | **1075** | (28.8) |  |  | **719** | (32.1) |  |  | **492** | (27.4) | **583** | (30.1) |  | **357** | (31.5) | **362** | (32.7) |
| Professional |  | **258** | (6.9) |  |  | **127** | (5.7) |  |  | **118** | (6.6) | **140** | (7.2) |  | **72** | (6.3) | **55** | (5.0) |
| Not working/Other |  | **69** | (1.8) |  |  | **67** | (3.0) |  |  | **39** | (2.2) | **30** | (1.5) |  | **35** | (3.1) | **32** | (2.9) |
|  |  |  |  |  |  |  |  |  |  |  |  |  |  |  |  |  |  |  |
| First language^3^ Marathi | **3733** | **2055** | (55.0) |  | **2239** | **1047** | (46.8) | <0.001^5^ |  | **1006** | (56.0) | **1049** | (54.2) |  | **526** | (46.4) | **521** | (47.1) |
| Hindi |  | **1327** | (35.5) |  |  | **912** | (40.7) |  |  | **627** | (34.9) | **700** | (36.2) |  | **475** | (41.9) | **437** | (39.5) |
| Other |  | **351** | (9.4) |  |  | **280** | (12.5) |  |  | **164** | (9.1) | **187** | (9.7) |  | **133** | (11.7) | **147** | (13.3) |
|  |  |  |  |  |  |  |  |  |  |  |  |  |  |  |  |  |  |  |
| Dietary intake  (frequency/week)^3^ | **3738** |  |  |  | **2243** |  |  |  |  |  |  |  |  |  |  |  |  |  |
| Milk <1 |  | **1880** | (50.3) |  |  | **1057** | (47.1) | 0.06^5^ |  | **913** | (50.8) | **967** | (49.8) |  | **522** | (46.0) | **535** | (48.3) |
| 1-6 |  | **1344** | (36.0) |  |  | **859** | (38.3) |  |  | **622** | (34.6) | **722** | (37.2) |  | **443** | (39.0) | **416** | (37.5) |
| ≥7 |  | **514** | (13.8) |  |  | **327** | (14.6) |  |  | **263** | (14.6) | **251** | (12.9) |  | **170** | (15.0) | **157** | (14.2) |
|  |  |  |  |  |  |  |  |  |  |  |  |  |  |  |  |  |  |  |
| GLV <1 |  | **907** | (24.3) |  |  | **540** | (24.1) | 0.02^5^ |  | **436** | (24.2) | **471** | (24.3) |  | **262** | (23.1) | **278** | (25.1) |
| 1-6 |  | **2743** | (73.4) |  |  | **1622** | (72.3) |  |  | **1319** | (73.4) | **1424** | (73.4) |  | **834** | (73.5) | **788** | (71.1) |
| ≥7 |  | **88** | (2.4) |  |  | **81** | (3.6) |  |  | **43** | (2.4) | **45** | (2.3) |  | **39** | (3.4) | **42** | (3.8) |
|  |  |  |  |  |  |  |  |  |  |  |  |  |  |  |  |  |  |  |
| Fruit <1 |  | **649** | (17.4) |  |  | **380** | (16.9) | 0.71^5^ |  | **302** | (16.8) | **347** | (17.9) |  | **187** | (16.5) | **193** | (17.4) |
| 1-6 |  | **2514** | (67.3) |  |  | **1501** | (66.9) |  |  | **1209** | (67.2) | **1305** | (67.3) |  | **765** | (67.4) | **736** | (66.4) |
| ≥7 |  | **575** | (15.4) |  |  | **362** | (16.1) |  |  | **287** | (16.0) | **288** | (14.8) |  | **183** | (16.1) | **179** | (16.2) |
|  |  |  |  |  |  |  |  |  |  |  |  |  |  |  |  |  |  |  |
| Meat and fish <1 |  | **1056** | (28.3) |  |  | **551** | (24.6) | 0.001^5^ |  | **497** | (27.6) | **559** | (28.8) |  | **288** | (25.4) | **263** | (23.7) |
| 1-6 |  | **2340** | (62.6) |  |  | **1443** | (64.3) |  |  | **1128** | (62.7) | **1212** | (62.5) |  | **720** | (63.4) | **723** | (65.3) |
| ≥7 |  | **342** | (9.1) |  |  | **249** | (11.1) |  |  | **173** | (9.6) | **169** | (8.7) |  | **127** | (11.2) | **122** | (11.0) |
|  |  |  |  |  |  |  |  |  |  |  |  |  |  |  |  |  |  |  |

^1^ Women who remained in the study include those who never became pregnant but stayed in the study until the end, and those who became pregnant and whose pregnancies were followed up; women who dropped out include those who migrated out of the study area, declined further follow-up, became sterilized, separated from their husbands, or died before the end of the trial. ^2^ Mean (SD) or ^3^ N (%) presented instead of median (IQR) for normally distributed or categorical variables respectively. Differences between groups were determined using t tests, except for ^4^ Mann Whitney U tests and ^5^ Chi-square or Fisher’s Exact tests. The p values for education and occupation were derived using the original 7 categories.

**Supplemental Table 3**: Newborn measurements according to allocation group and maternal body mass index

| **BIRTH MEASUREMENT** | **ALL** | | | **MATERNAL BMI**  **<18.6 kg/m^2^** | | | **MATERNAL BMI**  **18.6-21.8 kg/m^2^** | | | **MATERNAL BMI**  **>21.8 kg/m^2^** | | | **p for interaction^2^** |
| --- | --- | --- | --- | --- | --- | --- | --- | --- | --- | --- | --- | --- | --- |
|  | **Treatment** | **Control** | **p** | **Treatment** | **Control** |  | **Treatment** | **Control** |  | **Treatment** | **Control** |  |  |
| **Intention to treat analysis (women who started supplementation before their last menstrual period date)** | | | | | | | | | | | | | |
| N | **662** | **698** |  | **246** | **229** |  | **219** | **241** |  | **197** | **228** |  |  |
|  |  |  |  |  |  |  |  |  |  |  |  |  |  |
| Birthweight (g) | **2624** | **2598** | 0.22 | **2503** | **2526** |  | **2646** | **2612** |  | **2750** | **2654** |  | <0.001 |
|  | (390) | (395) |  | (366) | (375) |  | (380) | (403) |  | (389) | (396) |  |  |
| Crown-heel length (cm) | **47.5** | **47.6** | 0.42 | **47.0** | **47.3** |  | **47.7** | **47.8** |  | **48.1** | **47.8** |  | 0.002 |
|  | (2.4) | (2.3) |  | (2.3) | 2.4 |  | (2.2) | (2.2) |  | (2.5) | (2.3) |  |  |
| Head circumference (cm) | **33.1** | **33.1** | 0.74 | **32.8** | **32.9** |  | **33.2** | **33.2** |  | **33.4** | **33.4** |  | 0.04 |
|  | (1.3) | (1.3) |  | (1.4) | (1.4) |  | (1.2) | (1.2) |  | (1.3) | (1.3) |  |  |
| Chest circumference (cm)^1^ | **30.9** | **30.6** | 0.25 | **30.3** | **30.3** |  | **30.9** | **30.8** |  | **31.3** | **30.9** |  | 0.003 |
|  | (29.7, 31.9) | (29.6, 31.9) |  | (29.1, 31.4) | (29.3, 31.6) |  | (29.9, 32.0) | (29.7, 31.9) |  | (30.3, 32.5) | (29.9, 32.0) |  |  |
| Abdomen circumference (cm)^1^ | **28.5** | **28.4** | 0.41 | **28.1** | **28.0** |  | **28.6** | **28.6** |  | **29.2** | **28.6** |  | 0.02 |
|  | (27.3, 29.8) | (27.1, 29.8) |  | (26.9, 29.3) | (26.7, 29.4) |  | (27.5, 29.7) | (27.2, 30.0) |  | (27.6, 30.5) | (27.4, 29.9) |  |  |
| MUAC (cm) | **9.7** | **9.7** | 0.65 | **9.5** | **9.6** |  | **9.7** | **9.8** |  | **10.0** | **9.8** |  | <0.001 |
|  | (0.8) | (0.9) |  | (0.8) | (0.9) |  | (0.8) | (0.9) |  | (0.8) | (0.9) |  |  |
| Subscapular skinfold (mm)^1^ | **4.1** | **4.0** | 0.35 | **3.9** | **3.9** |  | **4.2** | **4.2** |  | **4.3** | **4.1** |  | 0.01 |
|  | (3.5, 4.7) | (3.5, 4.7) |  | (3.3, 4.4) | (3.3, 4.5) |  | (3.6, 4.8) | (3.5, 4.7) |  | (3.7, 5.0) | (3.5, 4.7) |  |  |
| Triceps skinfold (mm)^1^ | **4.1** | **4.0** | 0.23 | **3.9** | **3.9** |  | **4.1** | **4.1** |  | **4.2** | **4.0** |  | 0.02 |
|  | (3.5, 4.7) | (3.5, 4.7) |  | (3.3, 4.3) | (3.4, 4.4) |  | (3.6, 4.7) | (3.6, 4.9) |  | (3.8, 4.9) | (3.5, 4.6) |  |  |
| **Per protocol analysis (women who started supplementation >90 days before their last menstrual period date** | | | | | | | | | | | | | |
| N | **518** | **576** |  | **200** | **190** |  | **167** | **198** |  | **151** | **188** |  |  |
|  |  |  |  |  |  |  |  |  |  |  |  |  |  |
| Birthweight (g) | **2631** | **2583** | 0.046 | **2499** | **2507** |  | **2670** | **2591** |  | **2765** | **2651** |  | 0.001 |
|  | (394) | (404) |  | (371) | (381) |  | (390) | (417) |  | (377) | (401) |  |  |
| Crown-heel length (cm) | **47.6** | **47.6** | 0.90 | **47.0** | **47.3** |  | **47.8** | **47.6** |  | **48.2** | **47.9** |  | 0.004 |
|  | (2.4) | (2.3) |  | (2.4) | (2.4) |  | (2.2) | (2.2) |  | (2.6) | (2.4) |  |  |
| Head circumference (cm) | **33.1** | **33.1** | 0.87 | **32.8** | **32.9** |  | **33.3** | **33.1** |  | **33.4** | **33.4** |  | 0.09 |
|  | (1.3) | (1.3) |  | (1.4) | (1.4) |  | (1.2) | (1.2) |  | (1.3) | (1.3) |  |  |
| Chest circumference (cm)^1^ | **30.9** | **30.6** | 0.046 | **30.4** | **30.2** |  | **31.0** | **30.7** |  | **31.4** | **30.8** |  | 0.02 |
|  | (29.8, 32.0) | (29.5, 31.8) |  | (29.2, 31.4) | (29.3, 31.4) |  | (29.8, 32.0) | (29.5, 32.0) |  | (30.5, 32.6) | (29.9, 32.0) |  |  |
| Abdomen circumference (cm)^1^ | **28.5** | **28.3** | 0.25 | **28.1** | **27.9** |  | **28.6** | **28.5** |  | **29.2** | **28.6** |  | 0.08 |
|  | (27.3, 29.8) | (27.1, 29.8) |  | (27.0, 29.3) | (26.7, 29.4) |  | (27.4, 29.9) | (27.1, 30.0) |  | (27.7, 30.4) | (27.4, 29.9) |  |  |
| MUAC (cm) | **9.7** | **9.7** | 0.32 | **9.5** | **9.6** |  | **9.7** | **9.7** |  | **10.0** | **9.8** |  | 0.002 |
|  | (0.9) | (0.9) |  | (0.8) | (0.9) |  | (0.9) | (0.9) |  | (0.8) | (0.9) |  |  |
| Subscapular skinfold (mm)^1^ | **4.1** | **4.1** | 0.38 | **3.9** | **3.8** |  | **4.1** | **4.2** |  | **4.3** | **4.2** |  | 0.055 |
|  | (3.5, 4.7) | (3.4, 4.7) |  | (3.3, 4.5) | (3.3, 4.5) |  | (3.6, 4.7) | (3.5, 4.7) |  | (3.7, 5.0) | (3.5, 4.7) |  |  |
| Triceps skinfold (mm)^1^ | **4.1** | **4.0** | 0.33 | **3.9** | **3.9** |  | **4.1** | **4.2** |  | **4.2** | **4.0** |  | 0.03 |
|  | (3.6, 4.7) | (3.5, 4.7) |  | (3.4, 4.3) | (3.3, 4.4) |  | (3.6, 4.7) | (3.6, 4.9) |  | (3.8, 5.0) | (3.5, 4.7) |  |  |
|  |  |  |  |  |  |  |  |  |  |  |  |  |  |

Data presented are mean (SD) except for ^1^ (median (IQR)). ^2^ p value for interaction between supplementation group and maternal BMI.

**Supplemental Table 4: Effect of the intervention on birth weight according to different levels of compliance**

| **Mean no. of snacks consumed per week from 90 days prior to last menstrual period date until delivery** | **Intention to treat analysis** | | | | | |  | **Per protocol analysis** | | | | | |
| --- | --- | --- | --- | --- | --- | --- | --- | --- | --- | --- | --- | --- | --- |
|  | **Treatment** | | **Control** | | **Difference in birth weight (g)** | **p-value** |  | **Treatment** | | **Control** | | **Difference in birth weight (g)** | **p-value** |
|  | **Birthweight (g)** | **N** | **Birthweight**  **(g)** | **N** |  |  |  | **Birthweight (g)** | **N** | **Birthweight (g)** | **N** |  |  |
|  |  |  |  |  |  |  |  |  |  |  |  |  |  |
| **3 or more** |  |  |  |  |  |  |  |  |  |  |  |  |  |
| Lowest BMI | 2464 | 97 | 2541 | 137 | -77 | 0.12 |  | 2460 | 75 | 2515 | 107 | -55 | 0.33 |
| Middle BMI | 2646 | 92 | 2620 | 134 | +25 | 0.61 |  | 2657 | 68 | 2591 | 106 | +66 | 0.25 |
| Highest BMI | 2698 | 80 | 2683 | 122 | +15 | 0.79 |  | 2716 | 54 | 2651 | 96 | +65 | 0.34 |
|  |  |  |  |  |  |  |  |  |  |  |  |  |  |
| All | 2596 | 269 | 2612 | 393 | -16 | 0.59 |  | 2598 | 197 | 2583 | 309 | +15 | 0.67 |
|  |  |  |  |  |  |  |  |  |  |  |  |  |  |
| **2 or more** |  |  |  |  |  |  |  |  |  |  |  |  |  |
| Lowest BMI | 2483 | 154 | 2540 | 176 | -57 | 0.15 |  | 2479 | 120 | 2526 | 141 | -48 | 0.30 |
| Middle BMI | 2659 | 132 | 2626 | 177 | +33 | 0.44 |  | 2676 | 100 | 2601 | 139 | +75 | 0.14 |
| Highest BMI | 2717 | 134 | 2654 | 167 | +63 | 0.17 |  | 2736 | 97 | 2635 | 135 | +101 | 0.06 |
|  |  |  |  |  |  |  |  |  |  |  |  |  |  |
| All | 2613 | 420 | 2606 | 520 | +7 | 0.78 |  | 2620 | 317 | 2587 | 415 | +33 | 0.26 |
|  |  |  |  |  |  |  |  |  |  |  |  |  |  |
| **1 or more** |  |  |  |  |  |  |  |  |  |  |  |  |  |
| Lowest BMI | 2498 | 197 | 2525 | 208 | -28 | 0.44 |  | 2496 | 156 | 2504 | 169 | -8 | 0.85 |
| Middle BMI | 2661 | 178 | 2615 | 213 | +46 | 0.24 |  | 2698 | 133 | 2591 | 171 | +107 | 0.02 |
| Highest BMI | 2734 | 161 | 2657 | 197 | +77 | 0.07 |  | 2758 | 120 | 2647 | 160 | +111 | 0.02 |
|  |  |  |  |  |  |  |  |  |  |  |  |  |  |
| All | 2623 | 536 | 2598 | 618 | +25 | 0.28 |  | 2639 | 409 | 2579 | 500 | +59 | 0.02 |
|  |  |  |  |  |  |  |  |  |  |  |  |  |  |
| **All** | | | | | | | | | | | | | |
| Lowest BMI | 2503 | 246 | 2526 | 229 | -23 | 0.51 |  | 2499 | 200 | 2507 | 190 | -8 | 0.84 |
| Middle BMI | 2646 | 219 | 2612 | 241 | +34 | 0.35 |  | 2670 | 167 | 2591 | 198 | +79 | 0.07 |
| Highest BMI | 2750 | 197 | 2654 | 228 | +96 | 0.01 |  | 2765 | 151 | 2651 | 188 | +113 | 0.008 |
|  |  |  |  |  |  |  |  |  |  |  |  |  |  |
| All | 2624 | 662 | 2598 | 698 | +26 | 0.22 |  | 2631 | 518 | 2583 | 576 | +48 | 0.046 |
|  |  |  |  |  |  |  |  |  |  |  |  |  |  |

**SUPPLEMENTAL FIGURE LEGEND**

**Supplemental Figure 1:** Effect on birth weight according to categories of maternal BMI at differing levels of compliance (number of snacks eaten per week, on average, from 90 days before the last menstrual period date until delivery). A-D Intention to treat analysis; E-H Per protocol analysis
